# Development of antithrombotic nanoconjugate blocking integrin α2β1-collagen interactions

**DOI:** 10.1038/srep26292

**Published:** 2016-05-19

**Authors:** Chao Zhang, Lin Zhang, Youcai Zhang, Na Sun, Shaoyi Jiang, Timothy J. Fujihara, Yan Sun

**Affiliations:** 1 Department of Biochemical Engineering and Key Laboratory of Systems Bioengineering of the Ministry of Education, School of Chemical Engineering and Technology, Tianjin University, Tianjin 300072, People’s Republic of China; 2 School of Pharmaceutical Science and Technology, Tianjin University, Tianjin 300072, People’s Republic of China; 3 Departments of Chemical Engineering and Bioengineering, University of Washington, Seattle, WA 98192, USA

## Abstract

An antithrombotic nanoconjugate was designed in which a designed biomimetic peptide LWWNSYY was immobilized to the surface of poly(glycidyl methacrylate) nanoparticles (PGMA NPs). Our previous work has demonstrated LWWNSYY to be an effective inhibitor of integrin α2β1-collagen interaction and subsequent thrombus formation, however its practical application suffered from the formation of clusters in physiological environment caused by its high hydrophobicity. In our present study, the obtained LWWNSYY-PGMA nanoparticles (L-PGMA NPs) conjugate, with an improved dispersibility of LWWNSYY by PGMA NPs, have shown binding to collagen receptors with a *K*_d_ of 3.45 ± 1.06 μM. L-PGMA NPs have also proven capable of inhibiting platelet adhesion *in vitro* with a reduced *IC*_50_ of 1.83 ± 0.29 μg/mL. High inhibition efficiency of L-PGMA NPs in thrombus formation was further confirmed *in vivo* with a 50% reduction of thrombus weight. Therefore, L-PGMA NPs were developed as a high-efficiency antithrombotic nanomedicine targeted for collagen exposed on diseased blood vessel wall.

Interaction between platelets and collagen is crucial in the pathogenesis of thrombosis^1^,^2^,^3^,^4^,^5^, resulting in diseases of the cardiovascular system^5^,^6^,^7^,^8^. Many agents that inhibit the activation of platelets have been proven capable of inhibiting thrombus formation, but have the risk of causing unwanted bleeding^9^,^10^. For this reason it is urgent to develop new agents to prevent thrombus formation that do not have unwanted side effects. Collagen is a favorable target because it plays an essential role in platelet interaction with diseased blood vessels, constitutes the major protein in thrombotic plaques, and strongly contributes to lesion growth and arterial narrowing^11^. Blocking the collagen exposed on diseased blood vessel would prevent platelet adhesion while not affect the normal function of platelets, although its effect and safety was not fully accepted. For instance, increased embolization caused by targeting the collagen was reported^12^. In our previous work an effective inhibitor, LWWNSYY, was proposed to block the binding sites on collagen based on the naturally occurring interaction between integrin α2β1 (an important collagen receptor on platelets)^13^,^14^,^15^,^16^,^17^,^18^ and collagen. Significant inhibition of platelet adhesion by LWWNSYY was experimentally validated^19^,^20^, however the application of LWWNSYY was hindered by the formation of clusters in physiological environment caused by its high hydrophobicity. Improving the dispersibility of LWWNSYY was necessary for its practical application.

Conjugating the hydrophobic drugs to water soluble polymers or embedding them in macromolecules^21^,^22^ has been proven effective to improve the bioavailability. Ge *et al*.^23^ reported the encapsulation of hydrophobic anticancer drug in the hydrophobic core of a protein-polymer hybrid nanoparticles to enhance the anti-tumor activity. Manju *et al*.^24^,^25^,^26^ coupled curcumin onto hyaluronic acid to improve its aqueous solubility and stability. Sun *et al*.^27^ developed a novel spherical nanosilica matrix incorporated chitosan chains on the surface as carriers for poorly water soluble drug carvedilol to achieve a better bioavailability. Yang *et al*.^28^ synthesized a biocompatible polyurethane as carrier to achieve controlled release of hydrophobic drugs for a better drug utilization. Assali *et al*.^29^ reported a new carbon nanotubes named glyconanosomes, whose amphiphilic character allowed the water solubility of insoluble hydrophobic cargos such as a perylene-bisimide derivative, [60]fullerene, or the anti-carcinogenic drug camptothecin. Zhang *et al*.^30^ encapsulated hydrophobic anticancer drug in a cholic acid-modified dendritic multi-molecular micelle to achieve a better therapeutic efficacy. Chen *et al*.^31^ constructed a nano-prodrug to efficiently shield a hydrophobic antineoplastic agent doxorubicin in the core. The methods mentioned above are helpful to improve bioavailability of hydrophobic drugs, but are not appropriate for LWWNSYY because the embedded inhibitors could not reach the collagen receptors exposed on diseased blood vessel walls. For this reason immobilization of LWWNSYY on the exterior surface of nanoparticles was then considered. From the viewpoint of polymeric materials for biomolecule supports, poly(glycidyl methacrylate) nanoparticles (PGMA NPs) are of great interest with respect to protein carriers due to their high surface area and abundant surface functionalities (epoxides, hydroxyl groups, amines, and so on). The surface functionalities offer multiple attachment sites to ligands and drugs^32^. Such characteristics have been exploited for drug delivery applications^33^,^34^ and surface modification^35^,^36^,^37^. A diameter between 20 and 200 nm was chosen as an appropriate size for the carrier in consideration of splenic filtration and renal clearance^38^.

Therefore, a nanoconjugate was designed in which LWWNSYY was immobilized to the exterior surface of PGMA NPs with a diameter of ~100 nm^7^,^38^. The binding between LWWNSYY-PGMA nanoparticles (L-PGMA NPs) conjugate and collagen peptide was then investigated by isothermal titration calorimetry (ITC). Inhibition of platelet adhesion in the presence of L-PGMA NPs was examined *in vitro* by platelet solid-phase adhesion assays, and inhibition efficiency of L-PGMA NPs in thrombus formation was examined *in vivo* in a murine model of FeCl_3_-induced arterial thrombosis.

## Results

### Synthesis of L-PGMA NPs

**Figure 1:**
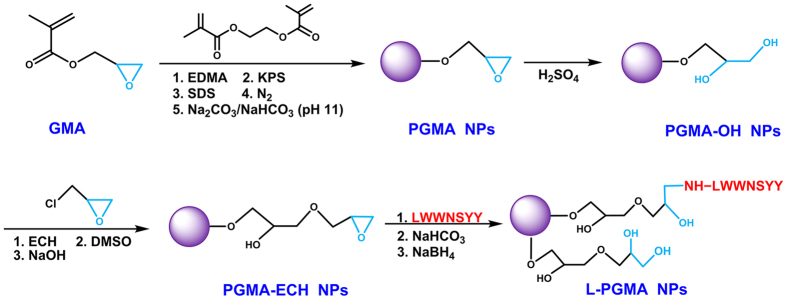


The synthetic procedure of L-PGMA NPs is shown in Fig. 1. PGMA NPs were obtained through the polymerization of glycidyl methacrylate (GMA) monomer. Ethylene glycol dimethacrylate (EDMA) was added as a cross-linking agent to improve the stability and intensity of PGMA NPs. Ring-opening reactions were then performed to obtain poly-glycerol methacrylate (PGMA-OH) NPs, which was further epoxy group-functionalized to extend the chain length to reduce the steric hindrance. The pendant epoxy groups on the surface of epoxy group-functionalized PGMA (PGMA-ECH) NPs could readily undergo ring-opening reactions with amine to achieve immobilization of LWWNSYY. Non-reacted epoxide groups were opened to reduce the interference in subsequent affinity binding.

**Figure 2:**
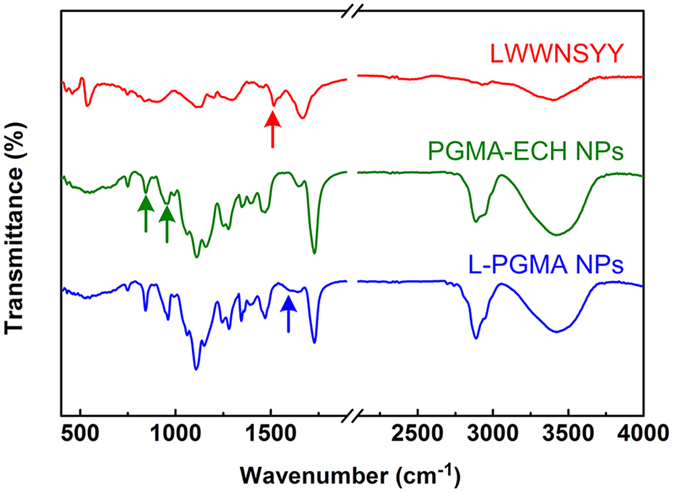
FTIR spectra of LWWNSYY (red), PGMA-ECH NPs (green) and L-PGMA NPs (blue). Arrows indicate the peaks of the amide bond (red), epoxide group (green), and -CNH- (blue).

The chemical conjugation of LWWNSYY onto the surface of PGMA-ECH NPs was examined using fourier transform infrared spectra (FTIR) spectroscopy (Fig. 2). LWWNSYY (Fig. 2, red curve) showed a peak at 1515 cm^−1^ corresponding to amine band (red arrow). PGMA-ECH NPs (Fig. 2, green curve) showed peaks centered at 844 cm^−1^ and 910 cm^−1^ corresponding to the epoxy groups (green arrows). After conjugated with LWWNSYY, the peaks corresponding to the epoxy group weakened and the stretching band of -CNH- (blue arrow) at 1580 cm^−1^ was observed in spectra of L-PGMA NPs (Fig. 2, blue curve). This indicates successful chemical conjugation of LWWNSYY onto the surface of PGMA-ECH NPs through the opening of epoxy group. UV-VIS spectra further confirmed formation of L-PGMA NPs (Fig. S1 in Supporting Information), with an absorption peak at 280 nm corresponding to the characteristic absorption of tryptophan residues in LWWNSYY. Using the absorption peak of L-PGMA NPs at 280 nm, the immobilized LWWNSYY was quantified to be 3.2 ± 0.2 mg in 100 mg of PGMA NPs, indicating significant decrease of the hydrophobic proportion after the conjugation to NPs. The formation of LWWNSYY clusters due to its high hydrophobicity was then expected to be inhibited.

**Figure 3:**
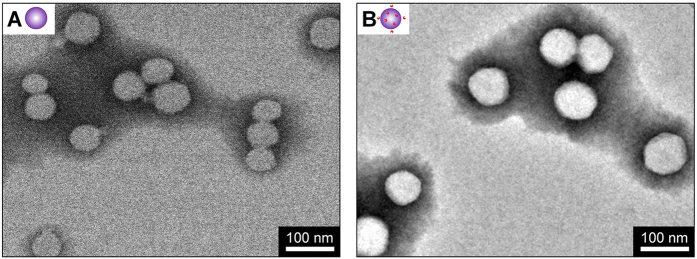
Transmission electron microscopy image of PGMA NPs (**A**) and L-PGMA NPs (**B**). Scale bar is 100 nm.

Particle size and zeta potential were measured by dynamic light scattering (DLS) in deionized water at room temperature, as shown in Table 1. The average hydrodynamic diameter of L-PGMA NPs was 106.6 ± 0.91 nm, slightly larger than that of PGMA NPs (102.2 ± 1.40 nm), but near the expected value of ~100 nm. Small polydispersity index (PDI) value was observed for both PGMA NPs (0.12 ± 0.04) and L-PGMA NPs (0.06 ± 0.02). A zeta potential of −27.2 ± 0.31 mV was observed for PGMA NPs (Table 1). Lower zeta potential of −32.9 ± 0.57 mV was observed after conjugation of LWWNSYY onto the surface of PGMA NPs. The monodispersed NPs were further confirmed by the Transmission electron microscopy (TEM) observations (Fig. 3).

### Inhibition efficiency of L-PGMA NPs in platelet adhesion *in vitro*

**Figure 4:**
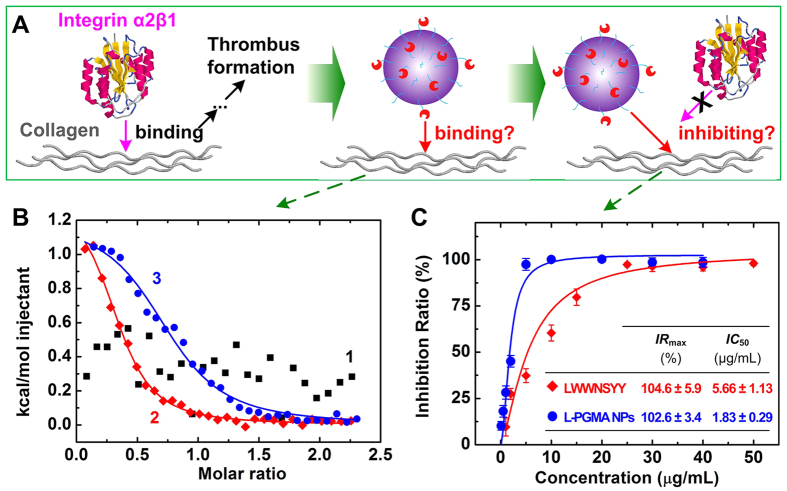
(**A**) Schematic diagram of examination on the inhibition efficiency of L-PGMA NPs in platelet adhesion *in vitro*. (**B**) The integral enthalpy profile of free LWWNSYY dissolved in normal saline titrated into collagen solution (#1), free LWWNSYY dissolved in normal saline containing 25% (v/v) ethylene glycol (EG) titrated into collagen solution (#2), and collagen titrated into L-PGMA NPs dissolved in normal saline (#3). The fit to the single-site binding model is shown by continuous curve. (**C**) Dose-response inhibition ratio of free LWWNSYY (red) and L-PGMA NPs (blue) on the platelet adhesion on collagen peptide. Continuous curves are the best fit obtained with the Hill equation. The obtained maximum inhibition ratio and the concentration required to inhibit half of the specific binding of inhibitors are shown in the inset. Data was presented as the mean ± S.D. from eight independent experiments (N = 8).

ITC experiments were performed to investigate the thermodynamic binding characteristics of L-PGMA NPs on the triple helical collagen peptide (Fig. 4A). The triple helix collagen peptide containing GFOGER recognition sequence was titrated with L-PGMA NPs. The data of LWWNSYY was also examined and used as control. The integral enthalpy profile is shown in Fig. 4B, and the thermodynamic binding constants are summarized in Table 2.

No thermodynamic binding constant was detectable for free LWWNSYY in normal saline (Fig. 4B, #1). LWWNSYY monomers formed clusters (see Fig. S2) covering their affinity sites, resulting in an irregular binding. Once 25% (v/v) EG was used to make a monodispersed LWWNSYY solution, a regular binding curve was observed (Fig. 4B, #2) and the thermodynamic binding constants could be calculated (Table 2). Negative Δ*G* confirmed favorable binding of LWWNSYY on collagen. The negative Δ*G* was contributed by a slightly positive Δ*H* and large positive *T*Δ*S*, indicating that the favorable binding was entropy-driven^39^,^40^, and that hydrophobic interaction played an important role in preventing binding. This was consistent with the results reported in previous work^20^. A regular binding curve was detected for L-PGMA NPs in normal saline (Fig. 4B, #3). Negative Δ*G* was also obtained, confirming its favorable binding on collagen (Table 2).

Platelet adhesion inhibition of L-PGMA NPs in physiological environment was examined by platelet solid phase adhesion assays (Fig. 4A). The inhibition ratio of free LWWNSYY and L-PGMA NPs at increasing concentrations (1–50 μg/mL for LWWNSYY and 0.1–40 μg/mL for L-PGMA NPs) on platelet adhesion on collagen is shown in Fig. 4C. The concentration of free LWWNSYY and L-PGMA NPs required to inhibit half of the specific binding (*IC*_50_) and the maximum inhibition ratio (*IR*_max_) are summarized and shown in the inset.

The inhibition ratio curves of both free LWWNSYY and L-PGMA NPs finally reached a plateau of 100%, but a much lower concentration of L-PGMA NPs was required than that of free LWWNSYY. *IC*_50_ of L-PGMA NPs was 1.83 ± 0.29 μg/mL, which decreased to 1/3 of that of free LWWNSYY (5.66 ± 1.13 μg/mL), indicating a better inhibition efficiency of L-PGMA NPs than LWWNSYY in platelet adhesion on collagen.

### Inhibition efficiency of L-PGMA NPs in thrombus formation *in vivo*

**Figure 5:**
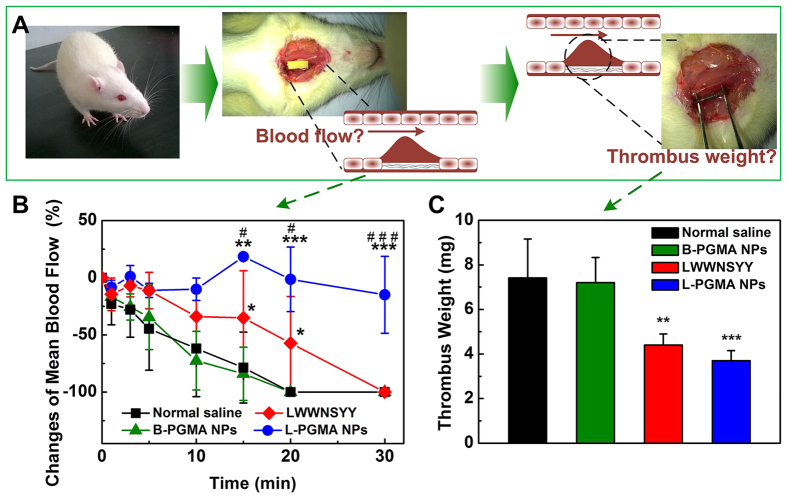
(**A**) Schematic diagram of examination on effects of inhibitors on carotid artery thrombus formation *in vivo*. (**B**) Percentage changes of mean blood flow, and (**C**) thrombus weights after 30 min injury of rats treated with normal saline (control, black), B-PGMA NPs (green), LWWNSYY (red) or L-PGMA NPs (blue). Data were presented as the mean ± S.D. from five independent experiments (N = 5). ^*^p < 0.05,^**^p < 0.01,^***^p < 0.001, compared to control. ^#^*p* < 0.05, ^###^*p* < 0.001, compared to LWWNSYY group.

To explore the potential of L-PGMA NPs as antithrombotic agents *in vivo* a murine model of FeCl_3_-induced arterial thrombosis was used (Fig. 5A).

The changes in mean blood flow and thrombus weights after various treatments are shown in Fig. 5B. In the control group (normal saline), a stop of blood flow was observed at about 20 min as a result of platelet occlusion (Fig. 5B), indicated by a mean blood flow decrease of 100%. In the presence of LWWNSYY the decrease rate of mean blood flow was slower as compared to control. Higher mean blood flow rate was observed at 20 and 25 min as compared to the control, and the time to occlusion was delayed to approximately 30 minutes. No occlusion was observed in L-PGMA NPs-treated rats at 30 min and mean blood flow rate at all time points was significantly higher compared to the control or LWWNSYY group. At the end of 30-min period L-PGMA NPs-treated rats were able to maintain mean blood flow at about 85% (*p* < 0.001, compared to control or LWWNSYY group) of initial blood flow. Thrombus weight of each group was also examined, as shown in Fig. 5C. In the control group, an intravascular thrombus of 7.41 ± 1.75 mg was obtained. Decrease of thrombus weight to 4.40 ± 0.50 mg (*p* < 0.01, compared to control) was observed when the rats were pretreated with LWWNSSYY, this accounts for nearly a 40% reduction compared to the control group. The smallest thrombus with a weight of 3.70 ± 0.45 mg (*p* < 0.001, compared to control) was obtained from the L-PGMA NPs treated group. Then reduced thrombus weight were observed in both LWWNSYY and L-PGMA NPs-treated groups, while no significant difference between these two groups was observed.

## Discussion

**Figure 6:**
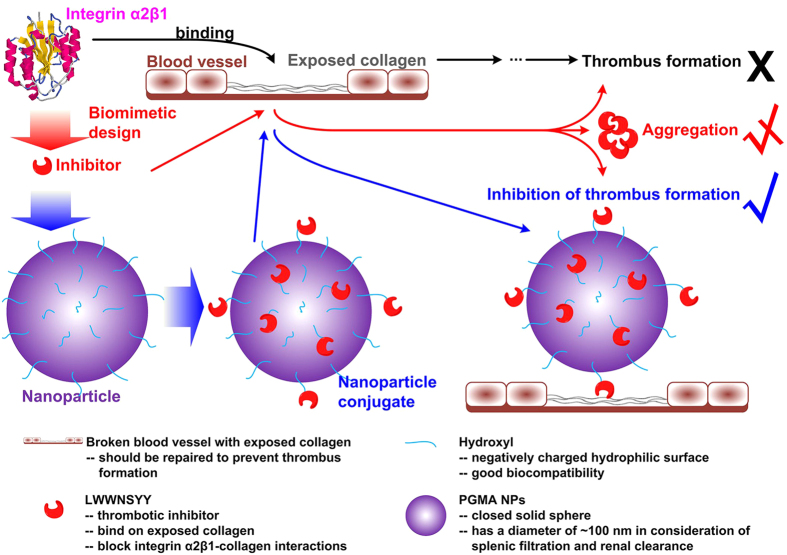
Schematic illustration of the design of nanoconjugate (L-PGMA NPs) to inhibit the thrombus formation. In the nanoconjugate, a designed biomimetic peptide LWWNSYY was immobilized to the surface of PGMA NPs to improve its bioavailability. L-PGMA NPs were expected to bind on the collagen exposed on diseased blood vessel wall to inhibit the thrombus formation.

In our previous work^19^,^20^, LWWNSYY was demonstrated as a high-efficiency inhibitor on the integrin α2β1-collagen interaction. However organic solvent was found necessary to achieve a regular binding due to high hydrophobicity of LWWNSYY. The examination of the molecular state of LWWNSYY in different solutions by fluorescent spectroscopy (Fig. S2) indicated that LWWNSYY aggregates to form clusters in a physiological environment, which limits its practical application. To overcome these obstacles a nanoconjugate was prepared in the present study by immobilization of LWWNSYY on the exterior surface of PGMA NPs, with a proposed mechanism shown in Fig. 6. A nanoparticle diameter of ~100 nm was chosen in consideration of splenic filtration and renal clearance^38^.

As shown in Fig. 2 and Table 1, successful chemical conjugation of LWWNSYY onto the surface of PGMA-ECH NPs was achieved. L-PGMA NPs with an average hydrodynamic diameter of 106.6 ± 0.91 nm was obtained, and the uniform size was indicated by small PDI value. Negative zeta potential of NPs provided enough surface charge to stabilize the particles against aggregation. Decreased zeta potential after conjugation of LWWNSYY onto the surface of PGMA NPs indicated increased surface charge and thus improved the dispersibility of NPs (further confirmed in the TEM observations in Fig. 3), which would be helpful for the long-term storage of L-PGMA NPs.

Inhibition efficiency of L-PGMA NPs in platelet adhesion was then examined *in vitro*. Regular binding curve was observed (Fig. 4B) once 25% (v/v) EG was used. Because organic solvent weakened the intermolecular hydrophobic interaction and thus prevented the aggregation of LWWNSYY monomer, and the exposed affinity sites made it capable of achieving a regular binding. However organic solvent is not appropriate to be used *in vivo*. After immobilization of LWWNSYY on the exterior surface of PGMA NPs, a regular binding curve was also detected (Fig. 4B) with a negative Δ*G*, confirming its favorable binding on collagen (Table 2). Similar thermodynamic binding constants as those of LWWNSYY in organic solvent were also obtained, indicating a spontaneous entropy-driven binding between L-PGMA NPs and collagen. In addition, negligible interactions between Blank-PGMA (B-PGMA) NPs and collagen were confirmed by an integral enthalpy of almost zero (see Fig. S3). Therefore, benefiting from the improved dispersibility of LWWNSYY by the immobilization of LWWNSYY on the surface of PGMA NPs, L-PGMA NPs is able to maintain the intrinsic binding characteristics of free LWWNSYY, but in normal saline without organic solvent (Figs 1 and 6). This modification has facilitated practical application in the physiological environment. Platelet adhesion inhibition of L-PGMA NPs in physiological environment was further examined by platelet solid phase adhesion assays (Fig. 4C). The results confirmed that the immobilization of LWWNSYY on the surface of L-PGMA NPs enhanced the effective concentration of inhibitor in physiological environment as a result of preventing LWWNSYY from forming clusters, which was consistent with the ITC results. Moreover, the binding characteristics of LWWNSYY were proven not affected after it was immobilized to the NPs.

The inhibition efficiency of L-PGMA NPs in thrombus formation was then examined *in vivo*. The FeCl_3_-mediated model of arterial injury was chosen because it was suitable to evaluate the inhibition of antithrombotic agents targeted on the binding between integrin α2β1 and collagen: FeCl_3_ has been shown to migrate through the endothelium by endocytic-exocytic pathways and cause endothelial denudation, leading to the exposing of collagen in the subendothelial matrix to the blood stream, and consequent activating of the coagulating system^41^,^42^,^43^. The dose of injected inhibitors had been proven safe to rats (Section S4) prior to the examination of inhibition efficiency. As shown in Fig. 5B, at the end of 30-min period L-PGMA NPs-treated rats were able to maintain mean blood flow at about 85% of initial blood flow and no occlusion was observed. Approximate 50% reduction of thrombus weight was observed in L-PGMA NPs treated group as compared to the control group and an approximate 15% reduction compared to LWWNSYY group (Fig. 5C). Herein, significant higher mean blood flow rate in L-PGMA NPs-treated rats was observed as compared to LWWNSYY group. However, the difference in clot weight was apparent small between these two groups. This could be attributed to the difficult operation and measurement error caused by the small absolute value of clot weight (with a magnitude of milligram), which resulted in the diminishing of the difference. Meanwhile, the liquid components in the sampled clot such as residual blood, caused further disturbance on the examination of clot weight. However, it can be seen that there was still a relative decrease of 15% of clot weight in L-PGMA NPs group, confirming the better inhibition efficiency achieved by L-PGMA NPs. Moreover, compared to the control group, rats treated with B-PGMA NPs showed a similar pattern of mean blood flow as well as thrombus weights, this indicates that B-PGMA NPs had no antithrombotic effect, and that LWWNSYY immobilized on the surface of L-PGMA NPs was the critical component for thrombosis inhibition. This is also consistent with the results of ITC (see Fig. S3). However, the aggregation of LWWNSYY due to its high hydrophobicity (Fig. S2) and consequent requirement of the assistance of organic solvent caused the limitation of its application, and also disturbance of its function, further emphasizing the advantages of L-PGMA. Therefore, both free LWWNSYY and L-PGMA NPs could inhibit thrombosis *in vivo*. L-PGMA NPs, however, possess a much better inhibition efficiency indicated by the smaller decrease of mean blood flow and smaller thrombus than that of free LWWNSYY. The results confirmed that L-PGMA NPs improved the dispersion of LWWNSYY through the immobilization of LWWNSYY on the surface of PGMA NPs, and then improved the binding of LWWNSYY, in effect producing an effective antithrombotic agent targeting the triple helix collagen. Its capability of altering platelet adhesion to other forms of collagen, or using human platelets could be examined in future. Meanwhile, based on the biomimetic design strategy presented herein, development of novel antithrombotic agent by mimicking other collagen receptors or targeting other factors in thrombus formation could be expected.

In conclusion, nanoconjugate L-PGMA NPs were designed and synthesized to inhibit thrombosis, where LWWNSYY was immobilized on the exterior surface of PGMA NPs to increase its dispersibility in aqueous solution. The obtained L-PGMA NPs proved capable of binding on collagen in the physiological environment by the ITC experimental results. Enhanced platelet adhesion inhibition by L-PGMA NPs was confirmed by platelet solid-phase adhesion assay, where *IC*_50_ of L-PGMA NPs decreased to 1/3 of that of free LWWNSYY. Moreover, high inhibition efficiency of L-PGMA NPs on thrombosis *in vivo* was confirmed by a murine model of FeCl_3_-induced arterial thrombosis. Therefore, L-PGMA NPs was successfully designed and demonstrated as a high-efficiency antithrombotic nanoconjugate with effective inhibition of thrombosis *in vivo*. Using of PGMA NPs herein provides an example for the effective improvement on the bioavailability of hydrophobic peptides in peptide-polymer conjugates. Furthermore, conjugating functional molecule to water soluble nanoparticles would be broaden to develop novel functional nanoconjugates. These findings would be helpful for the application of principles of nanotechnology in the development of multifunctional nanomedicines.

## Methods

Glycidyl methacrylate (97%) and ethylene glycol dimethacrylate (97%) were purchased from Sigma-Aldrich (St. Louis, MO, USA). Dimethy sulfoxide (DMSO), epichlorohydrin (ECH), EG and sodium borohydride (NaBH_4_) were of analytical grade from Guangfu Fine Chemical Research Institute (Tianjin, China). The triple helix collagen peptide, with an amino acid sequence of (GPO)_2_GFOGER(GPO)_3_, was purchased as HPLC-purified powder with a purity of >95% from ChinaPeptides (Shanghai, China). LWWNSYY was obtained as lyophilized powder with a purity of >95% from GL Biochem (Shanghai, China). Sheep blood containing 0.4% sodium citrate (w/v) was purchased from Ruite Bio-tec (Guangzhou, China). Bovine serum albumin (BSA) was purchased from Sigma (St. Louis, MO, USA). *ρ*-nitrophenylphosphate (PNPP) was purchased from Aladdin Industrial Corporation (Shanghai, China) with a purity of 98%. Sodium chloride (NaCl), tris (hydroxymethyl) aminomethane (Tris) and other reagents were of analytical grade from Sangon Biotech Co., Ltd. (Shanghai, China).

### Fluorescent spectroscopy

Prior to the experiment, LWWNSYY with different concentrations dissolved in normal saline or normal saline containing 25% (v/v) EG were equilibrated at 25 °C for at least 12 h. The fluorescent spectroscopy analysis was performed on a fluorescence spectrometer (Perkin Elmer LS-55, MA, USA) with excitation wavelength at 280 nm. The slit width of excitation was 10 nm, and that of emission was 8 nm. The emission spectra were collected within a range of 350–500 nm. The measurements were performed in triplicate, and the average value was used for the analysis.

### Preparation of PGMA NPs

10% GMA (v/v) and 0.2% EDMA (v/v) were added to a solution (10 mL) containing 2.5 mM Na_2_CO_3_, 2.5 mM NaHCO_3_, 0.2% (w/v) potassium persulfate and 0.55% (w/v) sodium dodecyl sulfate. After the mixture was flushed by nitrogen for 10 min to remove oxygen, polymerization reaction was performed under shaking (180 rpm) at 70 °C for 12 h. The non-reacted reactants were removed by dialysis against an excess amount of deionized water (changed three times a day) for three days at room temperature. The prepared PGMA NPs were stored at 4 °C for further use.

### Preparation of L-PGMA NPs conjugate

A reaction mixture of PGMA NPs and 0.5 M H_2_SO_4_ with a ratio of 1:2 (v/v) was prepared to open epoxide groups of PGMA NPs to obtain PGMA-OH NPs. The reaction was performed under shaking (180 rpm) at 60 °C for 3 h. The non-reacted reactants were removed by dialysis. Then, a reaction mixture of 10 mL PGMA-OH NPs, 10 mL DMSO, 5 mL ECH and 10 mL NaOH (1 M) was prepared to modify PGMA-OH NPs with epoxy group from ECH to obtain PGMA-ECH NPs. The reaction was performed under shaking (170 rpm) at 25 °C for 3 h. The non-reacted reactants were removed by dialysis and PGMA-ECH NPs was concentrated by PEG (Mw: 20000).

In preparation of L-PGMA NPs, 10 mL NaHCO_3_ (0.5 M) and 6 mg LWWNSYY were added into 5 mL PGMA-ECH NPs. The reaction was performed under shaking (170 rpm) at 25 °C for 12 h. Then, 56.81 mg NaBH_4_ (final concentration of 0.1 M) was added to the mixture to open the non-reacted epoxide groups. The reaction was performed under shaking (170 rpm) at 25 °C for 12 h. The non-reacted reactants were removed by dialysis. Appropriate ultrasonic treatment was used to avoid the aggregation of the nanoparticles and to make the nanoconjugate well dispersed.

### Fourier transform infrared spectroscopy

FTIR of the lyophilized samples (free LWWNSYY, PGMA and L-PGMA NPs) were determined by a TENSOR 27 instrument (Bruker Optics, Germany) in the range of 4000-400 cm^−1^.

### UV-VIS spectroscopy of L-PGMA NPs

UV-VIS spectra of L-PGMA NPs were obtained by Lambda 35 (Perkin Elmer, USA) UV-VIS spectrophotometer. Spectra were collected within a range of 200–600 nm. The nanoparticles without conjugated LWWNSYY were denoted as B-PGMA NPs. That is, the value of B-PGMA NPs with the same concentration was subtracted as background in analysis. Data were representative of three repeated experiments.

### Particle size and zeta potential

The size distribution and zeta potential of PGMA and L-PGMA NPs were measured with Zetasizer Nano (Malvern Instruments, UK). The samples were submitted to size or zeta analysis by injecting them into disposable DLS or zeta potential cuvettes at 25 °C. The results were reported as the average of three repeated experiments.

### Transmission electron microscopy

Morphology of PGMA and L-PGMA NPs was visualized by TEM using a JEM-2100F electron microscope (JEOL, Ltd., Japan) operated at an accelerating voltage of 200 kV. The samples (10 μL) for TEM were dropped on a carbon-coated copper grid (400-mesh) and air-dried for 5 min. The grid with nanoparticles was negatively stained with 2% (w/v) of phosphotungstic acid (pH 7.4) and air-dried.

### Isothermal titration calorimetry

The binding between collagen peptide and LWWNSYY in normal saline or normal saline containing 25% (v/v) EG was investigated following the procedure reported previously^20^, the concentration of collagen peptide in the cell was 80 μM, while the concentration of LWWNSYY was 1.0 mM. To investigate the binding between collagen peptide and L-PGMA NPs in normal saline, the concentration of L-PGMA NPs in the cell was 48 μM (in full text, the concentration of L-PGMA NPs represents the concentration of conjugated LWWNSYY on the surface of L-PGMA NPs in solution), while the concentration of collagen peptide was 600 μM. All samples were treated by 20 min degassing prior to the examination. Isothermal calorimetric titrations were performed in a titration mode with a 1.425 mL sample cell, using a VP isothermal titration calorimeter (MicroCal, Northampton, MA). 8 μL LWWNSYY was injected over 16 s for 30 times, with a spacing time of 350 s between injections. The reference power was set to 20 μcal/s, and the stirring speed was 307 rpm. To reserve the active triple helical form of collagen peptide^44^, the temperature in ITC cell was kept at 10 °C. In control, the titrant was injected into the buffer in the sample cell to obtain the heat of dilution, which was subtracted in following analysis. The titration between B-PGMA NPs and collagen was carried out to eliminate the interference of B-PGMA NPs. Titration data were analyzed using MicroCal Origin (Version 7.0). All possible binding sites were assumed to have the same binding energy. Consequently, the integrated enthalpy was calculated from the single-site binding model. Thermodynamics parameters, including *K*_d_, Δ*H, T*Δ*S*, and Gibbs free energy (Δ*G*) were calculated.

### Platelet solid-phase adhesion assays

Adhesion was determined colorimetrically^45^, following the procedure reported previously^46^,^47^. Anticoagulated sheep blood was obtained by the addition of 0.4% sodium citrate (w/v), allowing about five days for the transportation before the tests. Platelet-rich plasma (PRP) was obtained as the supernatant after centrifugation of the anticoagulated sheep blood at 180 g for 20 min at 4 °C. Then platelet was obtained as the precipitate after centrifugation of PRP at 1000 g for 10 min at 4 °C^12^, which was suspended in adhesion buffer (TBS containing 0.1% BSA) to prepare platelet suspension buffer for further use. Microtiter HB 96-well plates (Costar, Corning Inc., USA) were coated with 100 μL collagen peptide (100 μg/mL) dissolved in 0.01 M acetic acid for 4 h at 18 °C. Reagents without adhesion were discarded, and the wells were washed by 200 μL TBS (50 mM Tris-HCl, 140 mM NaCl, 2 mM Mg^2+^, pH 7.4) for three times. Then, wells were blocked for 1 h by 100 μL of TBS containing 50 mg/mL BSA. After three washes by 200 μL TBS, 100 μL LWWNSYY or L-PGMA NPs with different concentrations dissolved in normal saline was applied to each well and incubated at 18 °C for 1 h. 100 μL platelet suspension buffer were added to each well after three washes to remove non-reacted inhibitors, and plates were incubated at 18 °C for 1 h. Unbound platelets were then discarded, and the wells were washed as above. 150 μL lysis buffer (0.1 M citrate, pH 5.4, containing 0.1% Triton X-100 and 5 mM *p*-nitrophenyl phosphate) was added to each well. Reaction was terminated after 1 h by addition of 100 μL NaOH (3 M), and plates were read at 405 nm using an automated plate reader (ELX800, BioTek Instruments Inc., USA). The binding to collagen peptide in the absence of inhibitors was considered as positive control and set to 0% inhibition ratio while the well without addition of platelets was considered as negative control and set to 100% inhibition. The effect of binding to BSA or B-PGMA NPs was eliminated. According to the standard above, the absorption value was transformed to inhibition ratio. Data was presented as the mean ± S.D. from three independent experiments (n = 8). Dose-response inhibition ratio curves were fitted using the Hill equation^48^,^49^. The concentration of LWWNSYY or L-PGMA NPs required to inhibit half of the specific binding, *IC*_50_, and the maximum inhibition ratio, *IR*_max_, were calculated based on the Hill equation^50^.

### Animals

Adult SPF male Wistar rats weighing 180–220 g were purchased from HFK Bioscience Co. (Beijing, China), and housed in a standard temperature-, light-, and humidity-controlled animal enclosures at the institute of Radiation Medicine in Chinese Academy of Medical Sciences. All rats had free access to food and water, and were acclimated for at least 1 week. The Institute of Radiation Medicine Animal Care and Use Committee approved the protocols for the care and use of animals, and all animal procedures were carried out in accordance with the approved guidelines.

### Assay of carotid artery thrombus formation

FeCl_3_-induced arterial injury in rats was performed following the procedure reported previously^51^,^52^. Rats were randomly divided into 4 groups (N = 5) and fasted for 12 h before the surgery. They were anesthetized with 10% chloral hydrate (3 mL/kg, i.p.) and placed on a 37 °C warming pad throughout the experiment. The right carotid artery was exposed by blunt dissection with minimal blood loss, and a Doppler flow probe (Transonic T400, USA) was attached to the artery to monitor blood flow continuously. Fifteen minutes before induction of thrombosis^53^, the rats were intravenous injected with normal saline (5 mL/kg), LWWNSYY (0.625 mg/kg), L-PGMA NPs (0.625 mg/kg) or B-PGMA NPs (with the same nanoparticle concentration compared to L-PGMA NPs), respectively. Thrombus formation was subsequently induced by application of a filter paper (2.5 × 0.8 mm) saturated with 12% FeCl_3_ solution onto the exposed adventitial surface of the artery. After 20 min, the filter paper was removed, and the vessel was washed with normal saline. The blood flow was monitored continuously for 30 min after injury. At the end of 30-minute period, thrombus size was evaluated by the weight. Immediately after the experiment, the rats were sacrificed by cervical dislocation while still under deep anesthesia. Data are expressed as mean ± S.D. Data were analyzed by one-way ANOVA, followed by Duncan’s post hoc test. Statistical significance was set at p < 0.05.

## Additional Information

**How to cite this article**: Zhang, C. *et al*. Development of antithrombotic nanoconjugate blocking integrin α2β1-collagen interactions. *Sci. Rep.* **6**, 26292; doi: 10.1038/srep26292 (2016).

## Supplementary Material

Supplementary Information

## Figures and Tables

**Table 1:** Average Hydrodynamic Diameter and Zeta Potential of PGMA and L-PGMA NPs.

**Particle**	Hydrodynamic diameter (nm)*	Polydispersity index*	Zeta potential (mV)*
PGMA NPs	102.2 ± 1.40	0.12 ± 0.04	−27.2 ± 0.31
L-PGMA NPs	106.6 ± 0.91	0.06 ± 0.02	−32.9 ± 0.57

^*^Data was presented as the mean ± S.D. from three independent experiments (N = 3).

**Table 2:** Thermodynamic Binding Constants for inhibitors on Triple Helical Collagen Peptide.

**Conditions**^a^	***K***_**d**_ (μM)*	**Δ*****G*** (kcal/mol)*	**Δ*****H*** (kcal/mol)*	***T*****Δ*****S*** (kcal/mol)*
Free LWWNSYY in deionized water	ND	ND	ND	ND
Free LWWNSYY in 25% (v/v) EG	5.52 ± 0.92	−6.77 ± 0.04	1.53 ± 0.24	8.30 ± 0.28
L-PGMA in deionized water	3.45 ± 1.06	−7.09 ± 0.16	1.35 ± 0.21	8.44 ± 0.37

ND, not detectable.

^*^Data was presented as the mean ± S.D. from three independent experiments (N = 3).

^a^All the three conditions contained normal saline concentration.
