# Supplementary material for: Development of antithrombotic nanoconjugate blocking integrin α2β1-collagen interactions
Source: Sci Rep. 2016 May 19;6:26292. doi: 10.1038/srep26292 (PMC4872532; doi:10.1038/srep26292)
Supplement: Supplementary Information [file srep26292-s1.pdf]

Supplementary Materials

Development of antithrombotic nanoconjugate blocking integrin α2β1-collagen interactions

Chao Zhang[1], Lin Zhang[1,*], Youcai Zhang[2], Na Sun[1], Shaoyi Jiang[3], Timothy J. Fujihara[3], Yan Sun[1,*]

S1. UV-VIS Spectra of L-PGMA NPs and B-PGMA NPs

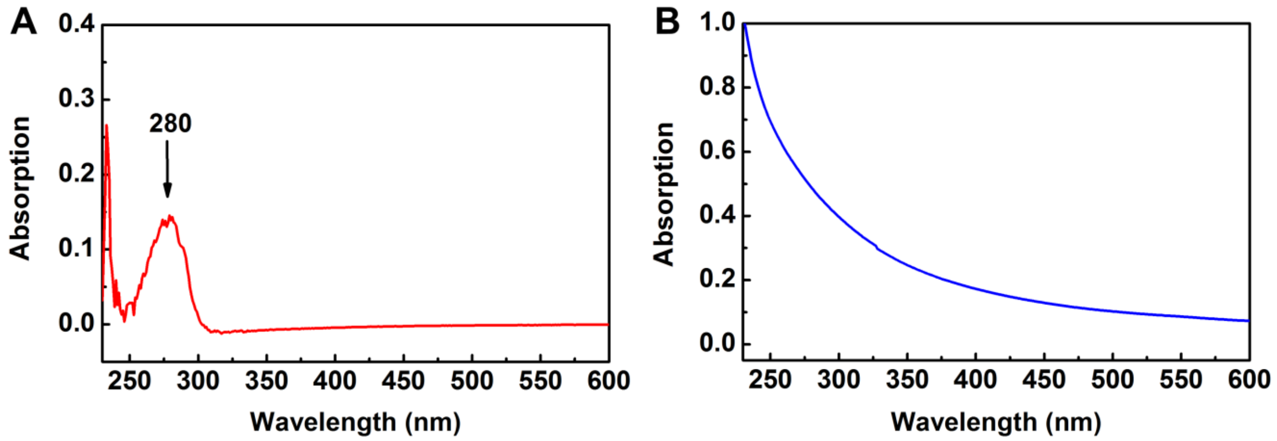

Figure S1. UV-VIS spectra of (A) L-PGMA NPs, the absorption of B-PGMA NPs were subtracted as background, and (B) B-PGMA NPs, the absorption of deionized water were subtracted as background.

S2. Molecular State of LWWNSYY in Different Solutions

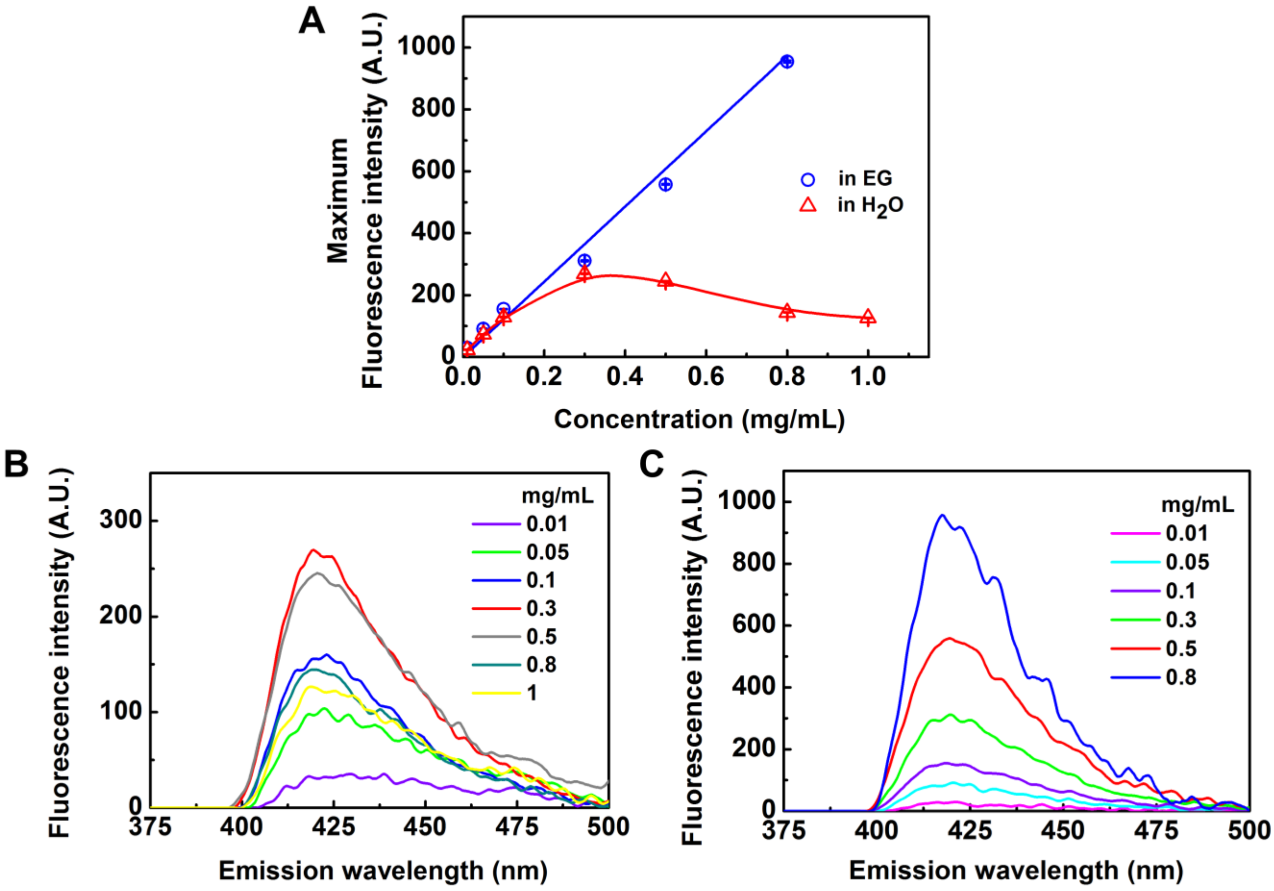

Figure S2. (A) The maximum fluorescence intensities of LWWNSYY with different concentrations in normal saline (red) and normal saline containing 25% (v/v) EG (blue). Data was presented as the mean ± S.D. from three independent experiments (N=3). The fluorescence spectra of LWWNSYY with different concentrations in (B) normal saline and (C) normal saline containing 25% (v/v) EG.

The molecular state of LWWNSYY in different solutions was investigated by fluorescent spectroscopy. According to literature,[1] fluorescent spectra obtained at an excitation wavelength of 280 nm were extensively used to detect the fluorescence of tryptophan residue and thus evaluate the exposure of the hydrophobic regions. Once LWWNSYY aggregated to

form clusters, fluorescence intensity would decrease due to the embedding of tryptophan residues. Then the maximum fluorescence intensities of LWWNSYY in different solutions were examined to evaluate the formation of LWWNSYY clusters, as shown in Fig. S2A. The fluorescent spectra are provided in Figs. S2B and C.

In normal saline containing 25% (v/v) EG, a linear increase of the maximum fluorescence intensity of LWWNSYY was observed (Fig. S2A, blue curve) at increasing concentrations (from 0.01 to 0.8 mg/mL), indicating that LWWNSYY was almost present as monomer in solution. In normal saline (Fig. S2A, red curve), lower maximum fluorescence intensity of LWWNSYY was observed as compared to that in ethylene glycol, indicating the aggregation of LWWNSYY leading to formation of clusters. The maximum fluorescence intensity of LWWNSYY first increased then decreased at increasing concentrations (from 0.01 to 1 mg/mL), because the aggregation became more serious when the concentration increased. The measurement reveals that without organic solvent to weaken the intermolecular hydrophobic interaction, LWWNSYY would aggregate into clusters in physiological environment.

S3. Interactions between B-PGMA NPs and collagen

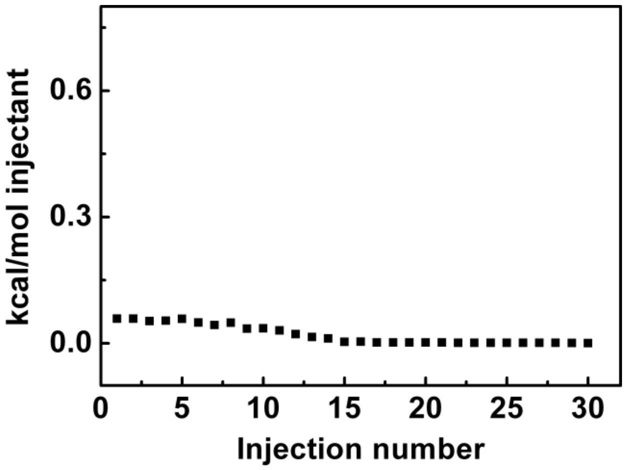

Figure S3. The integral enthalpy of the interactions between B-PGMA NPs and collagen in normal saline. The nanoparticle concentration of B-PGMA NPs in the cell was equal to that of L-PGMA NPs, while the concentration of collagen peptide was 600 μM.

S4. Toxicity Assay of Inhibitors

Eight specific pathogen free (SPF) ICR mice weighing about 18-22 g (HFK Bioscience Co., Ltd., Beijing, China) were randomly divided into two groups (N=4, half males and half females), and then treated with LWWNSYY or L-PGMA NPs (i.v., 2.5mg/10ml/kg) to investigate their toxicity, respectively. After treatment, animals were maintained under standard conditions with food *ad libitum* and maintained in a 12 h/12 h light/dark cycle at room temperature. Behavior, mortality and any other clinical signs of mice were observed and recorded for 7 days. The mice showed no abnormal clinical signs during the experiment, suggesting that both LWWNSYY and L-PGMA NPs had little toxicity at the dose of 2.5 mg/kg.

References

1.Bullock, A. N. *et al.*, Thermodynamic stability of wild-type and mutant p53 core domain. *P. Natl Acad. Sci. USA* **94**, 14338–14342 (1997).
